# A Study of the Bioactive Compounds, Antioxidant Capabilities, Antibacterial Effectiveness, and Cytotoxic Effects on Breast Cancer Cell Lines Using an Ethanolic Extract from the Aerial Parts of the Indigenous Plant *Anabasis aretioïdes* Coss. & Moq.

**DOI:** 10.3390/cimb46110735

**Published:** 2024-11-01

**Authors:** Salah Laaraj, Aziz Tikent, Mohamed Chebaibi, Khawla Bouaouda, Mohamed Bouhrim, Sherouk Hussein Sweilam, Rashed N. Herqash, Abdelaaty A. Shahat, Mohamed Addi, Kaoutar Elfazazi

**Affiliations:** 1 Agri-food Technology and Quality Laboratory, Regional Centre of Agricultural Research of Tadla, National Institute of Agricultural Research (INRA), Avenue Ennasr, Bp 415 Rabat Principal, Rabat 10090, Morocco; salah.laaraj@usms.ma; 2 Environmental, Ecological, and Agro-Industrial Engineering Laboratory, LGEEAI, Faculty of Science and Technology (FST), Sultan Moulay Slimane University (USMS), Beni Mellal 23000, Morocco; 3 Laboratoire d’Amélioration des Productions Agricoles, Biotechnologie & Environnement (LAPABE), Faculté des Sciences, Université Mohammed Premier, Bp 717, Oujda 60000, Morocco; tikent.aziz@ump.ac.ma; 4 Ministry of Health and Social Protection, Higher Institute of Nursing Professions and Health Techniques, Fez 30000, Morocco; mohamed.chebaibi@usmba.ac.ma; 5 Faculty of Science Ben M’sik, Laboratory of Biology and Health, University Hassan II of Casablanca, Casablanca 20650, Morocco; khawla.bouaouda-etu@etu.univh2c.ma; 6 Biological Engineering Laboratory, Faculty of Sciences and Techniques, Sultan Moulay Slimane University, Beni Mellal 23000, Morocco; mohamed.bouhrim@gmail.com; 7 Laboratoires TBC, Laboratory of Pharmacology, Pharmacokinetics, and Clinical Pharmacy, Faculty of Pharmaceutical and Biological Sciences, University of Lille, 3, rue du Professeur Laguesse, B.P. 83, F-59000 Lille, France; 8 Department of Pharmacognosy, Faculty of Pharmacy, Egyptian Russian University, Cairo-Suez Road, Cairo 11829, Egypt; s.sweilam@psau.edu.sa; 9 Department of Pharmacognosy, College of Pharmacy, King Saud University, Riyadh 11451, Saudi Arabia; rherqash@ksu.edu.sa (R.N.H.); ashahat@ksu.edu.sa (A.A.S.)

**Keywords:** southeastern Morocco, indigenous plant, *Anabasis aretioïdes*, antioxidant, antibacterial, anticancer

## Abstract

*Anabasis aretioïdes* contain numerous bioactive compounds that provide several advantages, including antioxidant, antibacterial, anticancer, neuroprotective, anti-inflammatory, and antidiabetic characteristics. This study aimed to make a hydroethanolic extract from the aerial part of the plant, analyze its biochemical compounds, and test its biological activities. From HPLC-DAD analysis, cinnamic acid, sinapic acid, and vanillin bioactives were found to be the main compounds in the extract. The spectrometric tests revealed that the extract was rich in flavonoids (8.52 ± 0.32 mg RE/100 g DW), polyphenols (159.32 ± 0.63 mg GAE/100 g DW), and condensed tannins (8.73 ± 0.23 mg CE/100 g DW). The extract showed significant antioxidant activity. There were strong correlations between the amount of flavonoid or polyphenol and the antioxidant assays, including ABTS, DPPH, β-carotene, and TAC. The extract also showed highly effective results against Gram-positive bacteria *Staphylococcus aureus* and *Enterococcus faecalis* as well as against Gram-negative bacteria *Escherichia coli* and *Pseudomonas aeruginosa*, and showed promising cytotoxicity against breast cancer cell lines MCF-7 and MDA-MB-231. The in silico modeling of the bioactive compounds contained in the extract illustrated their interaction mode with the active sites of particular target proteins, and it showed that rutin had the strongest effect on stopping NADPH oxidase enzyme, with a glide score of −6.889 Kcal/mol. Sinapic acid inhibited *E. coli* beta-ketoacyl-[acyl carrier protein] synthase (−7.517 kcal/mol), and apigenin showed high binding affinity to *S. aureus* nucleoside di-phosphate kinase, with −8.656 kcal/mol. Succinic acid has the strongest anticancer effect for caspase-3, with a glide score of −8.102 kcal/mol. These bioactive components may be beneficial as antioxidant and antibacterial applications in medicine, foods, natural cosmetics, and breast cancer prevention in the future. As a result, the use of this indigenous plant must be considered to maximize its value and preservation.

## 1. Introduction

Morocco possesses a significant diversity of plant species, estimated at 4500 taxa, encompassing 920 genera and 130 families. This botanical wealth can be utilized to discover novel and unique active compounds. It ranks first among the countries in the southern Mediterranean region in terms of its abundance of indigenous flora [1]. Out of the projected total of 1471 endemic species, 807 are found within the borders of Morocco, while 664 extend beyond Morocco’s geographical boundaries. These include Micronesian endemics, betico-rifain endemics, and Moroccan–Algerian endemics [1]. A few Moroccan–Algerian plants are native and have been investigated infrequently but possess genuine pharmacological characteristics. *Anabasis aretioïdes* Coss. & Moq. is one of these example plants, It belongs to the Chenopodiaceae family, which is indigenous to Morocco and Algeria [1]. In southeastern Morocco, this plant takes several colloquial names, such as Al-Sali’a and Bouamama [2]. The plant is extensively utilized in traditional medicine in Morocco and Algeria, where a decoction or infusion of the leaves and roots is created. It is commonly employed as an anti-rheumatic, a diuretic, a hypoglycemic, and a poison antidote. The root bark is utilized as firewood for burning [3,4]. The enduring adoption of traditional medicine can be ascribed to cultural and socioeconomic considerations, along with the inefficacy of certain conventional therapies [5]. The lack of actual medications and the capability of current antibiotic-resistant bacteria to withstand oxidative stress highlight the need for the creation of new medicinal substances derived from plants [6,7,8,9]. The therapeutic effects of *A. aretioïdes* Coss. & Moq. are attributed to its abundance of chemical constituents that exhibit significant biological features in laboratory settings, including antimitotic and antioxidant activity [10,11]. Multiple studies have shown that plants have antioxidant capacities mostly due to their phenolic components [12]. The pharmacological activities of these molecules have significant implications for human health, as they are capable of reducing inflammation, preventing allergies, combating bacterial and viral infections, and protecting against cancer. Additionally, they can aid in the treatment of cardiovascular diseases and facilitate blood vessel widening [13,14]. Furthermore, they can hinder the oxidative alteration caused by free radicals, scavenge oxygen, and break down peroxides using their antioxidant properties [15]. Currently, there is a focus on examining the efficacy of plants in traditional medicine due to their affordability and few side effects. Synthetic preservatives, which have been utilized in food for decades, may result in adverse health effects. Furthermore, the utilization of synthetic substances presents considerable disadvantages, including elevated costs, handling risks, issues regarding food residues, and threats to the human environment. Spices and herbs serve as viable alternatives to synthetic preservatives, functioning as natural, effective, and non-toxic agents. Spices and herbs, such as garlic, mustard, cinnamon, cumin, clove, thyme, basil, pepper, ginger, and rosemary, have served as food additives since antiquity, functioning as flavoring agents and natural preservatives. Several spices exhibit antibacterial properties against certain pathogens. Globally, the use of herbal medications is increasing as an alternative remedy for various health issues, such as cardiovascular illnesses, diabetes, hypertension, and specific types of cancer. In contrast to pharmaceuticals, herbal products lack regulation concerning their purity and efficacy. Herbal medications are classified as dietary supplements and are freely accessible on the market without a prescription [16].

Antimicrobial resistance poses a global challenge, underscoring the need for novel approaches from natural or synthetic origins. This underscores the importance of seeking innovative and safe therapeutic options [17,18]. The utilization of botanical extracts as a prospective remedy against pathogens and cancer cells finds substantiation in their antimicrobial agents and inherent antioxidants, which effectively counteract detrimental free radicals. Recognized for their medicinal attributes, plants exhibit the capability to avert and mitigate the adverse repercussions associated with conventional therapies [19,20]. Moreover, the integration of plant extracts with other therapeutic modalities holds considerable promise in the collective effort to address a spectrum of microorganisms and critical ailments, including cancer [21]. In this context, phytochemicals like flavonoids, phenolics, alkaloids, saponins, terpenes, and other compounds found in plant fractions and their extracts are recognized for their antioxidant, antimicrobial, and anti-cancer attributes [22,23,24]. Furthermore, the potential of secondary metabolites derived from medicinal plants to combat free radicals and prevent lipid peroxidation has been well studied. This protective effect against oxidative damage lowers the likelihood of undesirable cellular mutations [25,26].

Cancer is one of the world’s most serious health problems and the second-biggest cause of mortality after cardiovascular disease [27]. There is a suggestion that the socio-economic environment becomes biologically intertwined and correlates with distinct epigenetic markers that foster the progression of chronic health issues, including cancer [28]. Breast cancer constitutes one of the most prevalent malignancies and foremost contributors to female mortality [29,30]. Breast cancer is a notably heterogeneous disease, encompassing variations in patient tumors both within (intra-tumoral heterogeneity) and among individuals (inter-tumoral heterogeneity) [31,32,33]. Despite substantial strides in diagnosing and targeting breast cancer, it is gradually becoming a major type of persistent ailment. Treatment approaches and outcomes for breast cancer hinge on its subtypes, involving hormonal, radiotherapeutic, molecular, and chemotherapeutic interventions [34]. Nonetheless, challenges such as heightened tumor diversity, resistance to anti-cancer treatments (radiation therapy, chemotherapy, and endocrine therapy), and the substantial cost of existing therapies currently complicate effective breast cancer management [35,36].

The hydroethanolic extract from the aerial parts of the indigenous plant *Anabasis aretioides* in southeastern Morocco is under investigation to ascertain the veracity of its primary biological functions. This study seeks to demonstrate the aerial portion of this plant as a potential source of specific chemicals applicable in the treatment of various diseases. Our objective is to enhance the utilization and worth of these plants while simultaneously improving their status. The research indicated that *Anabasis aretioides* extract could be utilized to produce pharmaceuticals and natural compounds as alternatives to synthetic substances in the food business. It comprises a thorough process that incorporates spectroscopic analysis, high-performance liquid chromatography (HPLC), and in vitro assessments for antioxidant, antibacterial, and anticancer properties, along with in silico modeling. This methodology provides an in-depth comprehension of the bioactive properties of these extracts. We conducted various assays, providing a comprehensive assessment of the antioxidant potential. Furthermore, experimentation on certain cancer cell lines and bacteria broadens the scope of the research. The investigation of bioactive molecules in these extracts yields significant outcomes due to the comprehensive methodology, identification of specific bioactive substances, and applicability in both health and industrial domains. These extracts, however, underscore their therapeutic potential and are utilized in the culinary and pharmaceutical industries.

## 2. Materials and Methods

To accomplish the aforementioned objective, research was conducted to analyze the quantities of total polyphenols, flavonoids, and condensed tannins utilizing spectroscopic techniques. High-performance liquid chromatography (HPLC-DAD) analysis was also conducted to discover the quality profile of the bioactive components that may be correlated to their biological activities. Regarding that evaluation, the antioxidant potency was assessed using four different methods, including total antioxidant capacity, β-carotene bleaching assay, DPPH scavenging, and ABTS scavenging. Moreover, linear correlations are used to illustrate the connection between bioactive substances and antioxidant activity. Finally, we assessed the extract efficacy in eradicating four distinct bacterial strains and two distinct breast cancer cell lines (MCF-7 and MDA-MB-231). In some cases, the in silico method was employed to verify these biological activities and elucidate the mechanisms of action of certain bioactives.

### 2.1. Plant Origin and Extraction Process

**Figure 1:**
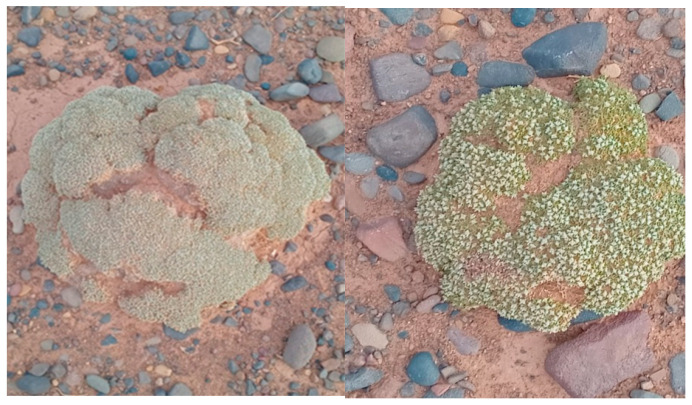
The original photos of *Anabasis arteriodes* Coss. & Moq. were taken from the study site in August 2023.

The aerial segments were collected from *A. aretioides* plants (Figure 1) flourishing in Figuig, Morocco (GPS coordinates of the harvest site: N 32°8′11.463″; W 1°13′37.173″) in August 2023. The voucher specimen of the plant was deposited at the plant section of Herbarium University Mohammed Premier Oujda, Morocco (HUMPO 43). In the beginning, these segments were processed in a commercial blender. Next, they were “macerated” in a process called “solid–liquid extraction,” which involved mixing 10 g of the powder that was made with 50 mL of 95% ethanol. Employing a vacuum pump, the mixture underwent filtration, subsequently culminating in the extraction process. This extract, called *Anabasis aretioïdes* hydroethanolic extract (AAE), was kept at −4 °C until it was needed. To do this, the solvent was evaporated in an electrical rotary evaporator at 250 bar pressure, 60 °C temperature, and 150 rpm.

### 2.2. Bioactive Compound Measurements

The total amount of polyphenols in the AAE was determined using the Folin–Ciocalteu method in accordance with the established protocol given by Rafique et al. (2023) [37]. Initially, 200 µL of Folin–Ciocalteu reagent and 100 µL of the extract (2 mg/mL) were mixed together in 2 mL of distilled water to generate an extract solution. The mixture was then mixed with 1 milliliter of a 15% sodium carbonate solution. After that, the resultant solution was kept at room temperature for two hours in a dark spot. The absorbance in a spectrophotometer at a specified wavelength of 765 nm was measured. Gallic acid was used to create a calibration curve with a concentration range of 0–0.1 mg/mL. Gallic acid equivalents, or mg GAE/100 g DW, are units of measurement used to express the overall amount of phenolics.

To discover the quantity of flavones and flavanols in the AAE, it was put through a colorimetric test with aluminum chloride (AlCl_3_), following the steps explained by Frond et al. (2019) [38]. In summary, 500 microliters of each extract at a concentration of 2 milligrams per milliliter were combined with 1.5 mL of methanol. Subsequently, 100 µL of AlCl_3_ solution with a concentration of 10% and 100 µL of potassium acetate solution with a concentration of 1 M were introduced. Then, by adding distilled water, the total capacity was increased to 2.8 mL. Following a half-hour incubation period at room temperature without any illumination, the absorbance was measured at 415 nm in comparison to a reference sample.

The condensed tannin content of the AAE was measured using the vanillin method, as described by Mohti et al. (2020) [39]. In this stage, 1.5 mL of vanillin (4% methanol) were combined with 50 microliters of the extract solution. The mixture was kept smooth by stirring/vortexing slightly. Subsequently, 750 microliters of strong hydrochloric acid (HCl) were introduced. Under dark conditions, the mixture was then incubated at ambient temperature for 20 min. Afterwards, the amount of light absorbed by the combination was measured at a wavelength of 500 nm. We measured the amount of condensed tannins in mg CE/100 g DW. To make a calibration curve, we used catechin at concentrations of 0 to 5 mg per milliliter. In order to guarantee precise and consistent outcomes, all measurements were conducted three times.

### 2.3. Analysis of Phenolic Compounds (HPLC-DAD)

The plant ethanolic extracts were evaluated using real and sophisticated analytical methods. One of the instruments used was an HPLC/DAD system developed by the Waters Corporation, Milford, MA, USA. This system combines a diode array detector with HPLC. The Empower program was used to further analyze the data obtained from this study [40]. Using automatic injection equipment, 20 µL of the samples were introduced into a Zorbax XDB-C18 column, manufactured by Agilent Technologies, Santa Clara, CA, USA (5 µm porosity, 250 × 4.6 mm), and 0–25 min at 20% B, 25–30 min at 100% B, and 30–35 min at 20% B made up the elution gradient. Using an Agilent ChemStation HPLC system operating at a flow rate of 1 mL/min, the collected signals were merged. Two mobile phases—A, consisting of water with 0.5% phosphoric acid, and B, consisting of methanol—were used to elute the material. A constant temperature of 40 °C was maintained throughout the separation process. At a wavelength of 280 nm, spectrophotometry was used to conduct the measurements. We contrasted the UV spectra and retention times of the identified peaks with those of a reference solution (5 mg/mL) that underwent analysis under the same conditions and with the same column. This assisted us in identifying the compounds contained in the ethanolic extract [40].

### 2.4. Antioxidant Activity

#### 2.4.1. DPPH Assay

The antioxidant potential of AAE was evaluated by means of a modified DPPH technique, in accordance with established protocol [41,42]. The DPPH-MeOH solution was created by dissolving 2 mg of DPPH in 100 mL of methanol. A series of AAE solutions were created at varying concentrations of between 5 and 500 µg/mL. Next, 2.5 mL of the DPPH mixture were added to each of the individual AAE solutions, and the total volume was adjusted to 3 mL. After a 30 min incubation period at room temperature, the combination’s absorbance was determined at 517 nm relative to a blank. The formula used to determine the amount of DPPH free radical scavenging activity was as follows:

$$Free\;Radical\;Scavenging\left(\%\right)=\left[\left(\frac{A_{blank}-A_{sample}}{A_{blank}}\right)\right]\times 100$$

The absorbance of the control reaction, which consisted of all reagents except for the extract, was designated as “A blank,” whereas the absorbance of the extract at varying concentrations was designated as “A sample.” A standard technique for determining the IC_50_ entails creating a graph that plots the inhibition percentage versus the extract concentrations. It was critical to use ascorbic acid as a positive control.

#### 2.4.2. β-Carotene Bleaching Assay

To assess the antioxidant activity of AAE, we used the β-carotene test, which measures the extent of bleaching. We used a modified version of the method published in earlier research by Kandsi and Elbouzidi et al. (2022) [24] and Rădulescu et al. (2021) [43]. At first, a solution containing 2 mg of β-carotene in 10 mL of chloroform was created. This solution was then combined with a solution containing 20 mg of linoleic acid and 200 mg of Tween-80. The chloroform was extracted using a rotavapor at a temperature of 40 °C, and 100 mL of distilled water were introduced with vigorous agitation. The obtained samples were evenly divided into three identical sets over a 96-well plate and kept in a place with restricted light exposure at a temperature of 25 °C for a duration of 30 min. After adding the AAE solution (t0), the samples were immediately examined using spectrophotometry at a wavelength of 470 nm. Following a two-hour incubation period (t1), a second measurement was taken, and both levels were compared to a baseline measurement that contained all of the AAE solution’s ingredients except for β-carotene. Butylated hydroxytoluene (BHT) served as the experiment’s control. To obtain dependable and precise outcomes, the residual color % was ascertained by applying the subsequent formula:

$$Residual\;color\left(\%\right)=\left[\left(\frac{Initial\;OD-Sample\;OD}{Initial\;OD}\right)\right]\times 100$$

#### 2.4.3. ABTS Scavenging Activity Assay

A customized protocol based on the technique outlined by Nakyai et al. (2021) was used to examine AAEE’s capacity to eliminate ABTS radicals [44]. To create the ABTS^•+^ radical cation, 2.45 mM potassium persulfate were combined with the ABTS solution. After that, the combination was kept for 16–18 h at room temperature in a dark place. At a wavelength of 750 nm, the resultant solution was further diluted with ethanol till its absorbance reached 0.70 ± 0.02. L-ascorbic acid served as a favorable control. To perform the ABTS assay, 200 µL of the previously diluted ABTS^•+^ solution were combined with a volume of 20 µL of the test material. Following a 10 min dark incubation period at room temperature, the resulting mixture’s absorbance was measured using a microplate reader at a wavelength of 734 nm. Analogous to the DPPH experiment, the ABTS radical cation elimination capacity was assessed using the same approach.

#### 2.4.4. Total Antioxidant Capacity

The phosphor–molybdenum technique was utilized to ascertain the antioxidant activity in the specimen [45]. The sample extract/standard solution was mixed with a reagent solution comprising 0.6 M sulfuric acid, 28 mM sodium phosphate, and 4 mM ammonium molybdate in accordance with the prescribed procedure. After that, the mixture was incubated for 90 min at 90 °C before being allowed to cool to ambient temperature. Using a standard curve created using ascorbic acid, the absorbance of the resultant solution was measured at 695 nm and reported in terms of ascorbic acid equivalents [46]. All required materials were present in the blank solution that did not contain the test sample. Triplicate measurements were carried out to ensure that the results were accurate and consistent.

### 2.5. Antibacterial Activity

#### 2.5.1. Bacterial Strains and Growth Conditions

This study was carried out against four different bacterial strains. Two types of Gram-positive bacteria were used: *Staphylococcus aureus* (ATCC 6538) and *Enterococcus faecalis* (ATCC 29212), and two types of Gram-negative bacteria: *Escherichia coli* (ATCC 10536) and *Pseudomonas aeruginosa* (ATCC 15442). The bacterial strains were cultivated on Luria–Bertani–Agar (LBA) medium and then placed in an incubator at a temperature of 37 °C for a period of 24 h. A UV-visible spectrophotometer set to 620 nm was used to measure and set the bacterial concentration to 10^6^ cells/mL before the AAE extract was applied.

#### 2.5.2. Disc Diffusion Method

Following the guidelines set by the National Committee for Clinical Laboratory Standards (NCCLS) [47], the disc diffusion method was used to test how well AAE killed mycobacteria as an antibacterial agent. The Wayne approach is efficacious for assessing the substance’s capacity to impede mycobacterial proliferation [48]. A suspension of 10^8^ microorganisms per milliliter in physiological saline was introduced onto Mueller–Hinton agar plates [49]. A paper disk devoid of any content, with a volume of 10 µL of AAE, was subsequently positioned on the surface of the grown medium. The plates were thereafter placed in an incubator set at a temperature of 37 °C for a duration of 24 h. The inhibition diameters were then measured in millimeters with a ruler. The experiment was performed three times, using imipenem (10 µg/disc) or amoxicillin (25 µg/disc) discs as the positive control.

#### 2.5.3. Determination of the MIC and the MBC

The minimum inhibitory concentration (MIC) was determined in order to evaluate the efficacy of chemicals that combat microorganisms. The research described in this article utilized the resazurin micro-titer test to determine the minimum inhibitory concentration (MIC) of the *A. aretioides* extract. This test utilizes a color-changing material known as resazurin, which is chemically reduced by active cells, resulting in a change in hue from blue to pink. The antimicrobial agent was introduced at varying doses into each well of a 96-well microplate, followed by the addition of a standardized inoculum of the bacteria under investigation. The microplates were thereafter placed in an incubator at a temperature of 37 °C for a duration of 24 h. Following this, resazurin was added to each well, as described in the reference Balouiri et al. (2016) [50]. The plates were incubated for a further 4–6 h until a noticeable shift in color occurred. The minimum inhibitory concentration (MIC) was established as the lowest concentration of the antimicrobial drug that caused no discernible color change, demonstrating the complete absence of live bacteria. Controls were used to ensure the outcomes were precise.

The MBC was measured by inoculating a sample from the negative wells onto Mueller–Hinton agar medium plates. The plates were thereafter placed in an incubator set at a temperature of 37 °C for a duration of 24 h. As illustrated in the reference, the minimum bactericidal concentration (MBC) of the extract that did not lead to the growth of bacteria was identified. The experiment was replicated three times to verify its repeatability.

### 2.6. Cytotoxicity Against Breast Cancer Cell Lines

#### 2.6.1. Cell Culture

Two estrogen receptors of the breast cancer cells were employed in this study: estrogen receptor-positive (MCF-7 cells) and estrogen receptor-negative (MDA-MB-231 cells). The cells were grown in Dulbecco’s Minimum Essential Medium (DMEM) supplemented with 10% fetal bovine serum (FBS) and 50 µg/mL gentamicin. To maintain the viability of the cells, the culturing was carried out in a humid atmosphere at 37 °C with 5% CO_2_. To guarantee continued proliferation, the cells were transmitted in 25 cm^2^ tissue culture flasks. The study utilized cells in the rapid growth phase to examine viability.

#### 2.6.2. Cell Viability by MTT Assay

In order to assess the potential of AAE to impede the growth of cancer cells, we employed the MTT test, as outlined in the references Chaudhary et al. (2015) [45] and Elbouzidi et al. (2023) [51]. MCF-7 and MDA-MB-231 cells were placed in 96-well plates at a concentration of 104 cells per well in 100 µL of media. They were then given 24 h to attach to the plates. Varying amounts of AAE were created by dissolving it in 0.1% DMSO and then diluting it with a medium. Subsequently, the cells were subjected to different doses of AAE for a duration of 72 h. The cells in the control group were only exposed to a medium containing 0.1% DMSO. After 200 µL of culture medium were added instead of the medium, 20 µL of MTT reagent (5 mg/mL MTT in PBS) were added, and the mixture was left to sit at 37 °C for 4 h. Following the removal of the medium, 100 µL of DMSO were introduced. The absorbance at 540 nm was then determined using a microplate reader (Synergy HT Multi-Detection Microplate Reader, Bio-Tek, Winooski, VT, USA). This measurement was utilized to compute the percentage of cell viability; the study evaluated the effect of AAE on cell viability by quantifying absorbance using the provided equation.

$$Cell\;viability\left(\%\right)=100-\left[\left(\frac{A_{0}-A_{t}}{A_{0}}\right)\times 100\right]$$

A_0_ = absorbance of cells treated with 0.1% DMSO medium, and A_t_ = absorbance of cells treated with AAE at various concentrations. A negative control group was given 0.1% DMSO in the medium, and GraphPad Prism 8.01 software was used to calculate IC_50_ values, with cisplatin as the standard.

### 2.7. Molecular Docking Study

The AAE was tested for its antioxidant, antimicrobial, and anticancer capabilities. The Schrodinger Suite’s Maestro 11.5 software was used to perform the molecular docking analysis. This study set out to discover how the compounds contained in the hydroethanol extracts from the *Anabasis aretioïdes* interact with the active sites of particular target proteins. These proteins include NADPH oxidase, beta-ketoacyl-[acyl carrier protein] synthase, nucleoside diphosphate kinase, sterol 14-alpha demethylase (CYP51), and caspase-3.

#### 2.7.1. Protein Preparation

The crystal structures of the target proteins, namely, NADPH oxidase (PDB ID: 2CDU), beta-ketoacyl-[acyl carrier protein] synthase from *Escherichia coli* (PDB ID: 1FJ4), nucleoside diphosphate kinase from *Staphylococcus aureus* (PDB ID: 3Q8U), sterol 14-alpha demethylase (CYP51) from *Candida albicans* (PDB ID: 5FSA), and caspase-3, were obtained from the RCSB database. The process of protein preparation was carried out using the Protein Preparation Wizard of Maestro 11.5. This involved a series of phases, including preprocessing, refining, and reduction. Hydrogen atoms were introduced, and hydroxyl groups, water molecules, and amino acids were rearranged to correct structural defects such as atom overlap or absence. The proteins were subsequently modified delicately to enhance their structural characteristics [52,53,54].

#### 2.7.2. Ligand Preparation

For ligand generation, the Schrödinger suite’s Ligprep wizard in Maestro 11.5 was used. This requires utilizing the OPLS3 force field to reduce structures, add hydrogen atoms, resolve bond length and angle issues, and transform 2D structures into 3D. The ionization states were fine-tuned while maintaining chirality [55].

### 2.8. Statistical Analysis

Microsoft Office Excel 2021 was utilized to determine the correlation coefficients (R^2^) for the spectrophotometric experiments. Statistical analysis was performed using IBM SPSS Statistics V21.0 software for descriptive statistics, for comparing averages with ANOVA one-way analysis of studied characteristics by post hoc (Tukey’s) test at the 5% threshold, for bivariate Pearson correlation analysis with a significance level of *p* < 0.01, and for Principal Component Analysis (PCA).

## 3. Results and Discussion

### 3.1. Spectrophotometric Analysis of Bioactive Components in AAE

Table 1 shows the spectrophotometric result of the studied bioactive compounds: total polyphenol content (TPC), total flavonoid content (TFC), and condensed tannin content (TCT) of AAE.

The total polyphenol was 159.32 ± 0.63 mg GAE/100 g DW. The findings of our study align well with those of Senhaji et al. (2020), as we observed similar amounts ranging from 65 to 178 mg GAE/100 g for aqueous extracts [1]. Nevertheless, the TPC levels discovered by Berrani et al. (2019) and El-Haci et al. (2013) were much higher, measuring 463 mg GAE/100 g and 231.85 mg GAE/g, respectively [10,11]. Polyphenolic compounds can protect cellular components from damage caused by free radicals and hinder the development of degenerative illnesses, including cancer and cardiovascular ailments [56].

The total flavonoid content was 8.52 ± 0.32 mg RE/100 g DW. Flavonoids are a very significant category of polyphenols known for their potent antioxidant properties. Additionally, they have shown pharmacological action, including antimicrobial properties, anti-inflammatory properties, and inhibition of platelet aggregation [57].

The condensed tannin content was 8.73 ± 0.23 mg CE/100 g DW. Our finding was lower than that of Berrani et al. (2019) (17 mg RE/100 g and 256 mg CE/100 g, respectively) [11]. Tannins, categorized as condensed tannins and hydrolyzable tannins, are the primary bioactive substances responsible for the astringent properties of several plants, particularly medicinal ones. This directly impacts their nutritional quality and taste [57].

The composition of natural substances is significantly influenced by multiple elements, such as environmental circumstances, including the time of harvest, biotic and abiotic stress, storage conditions, extraction processes, and other associated aspects. These factors can significantly influence the quantities of secondary metabolites, particularly phenolic compounds. These molecules play a crucial role in various drug-related activities, as they provide significant health benefits due to their antioxidant properties [58,59]. Different factors like species and physiological state, and outside factors like season, climate, and temperature, can change the number of polyphenols in *Anabasis aretioides* [60]. The variability may also be influenced by the choice of maceration extraction method (warm or cold) and the extraction solvent. To enhance the efficiency of polyphenol extraction, it is imperative to meticulously select a suitable solvent [61].

### 3.2. Antioxidant Activity of AAE

The human body relies on antioxidant activity to safeguard cells against the harmful effects of free radicals, which are generated during several oxygen-dependent processes and contribute to oxidative damage [62]. The antioxidant activity of AAE was evaluated using four separate methods (Table 2). The examination of the findings demonstrated considerable antioxidant efficacy.

In terms of DPPH IC_50_, AAE antioxidant activity was measured at 51.28 ± 0.91 mg Asc. equivalence per milliliter. This obtained result is higher than the DPPH (IC_50_ = 0.61 ± 0.01 μg/mL) found in the study by Assia Berrani et al. (2018) in a methanolic extract of the aerial part of *A. aretioïdes* [11]. Souad Senhaji et al. (2020) found values of DPPH IC_50_ ranging between 52.91 ± 0.24 and 3704.33 ± 5.97 μg/mL as a result of the antiradical activities of aqueous and organic extracts of the aerial part of *A. aretioïdes* [1]. The antioxidant potency of AAE in the β-carotene bleaching assay measured 4.44 ± 0.21 mg BHT equivalent/mL. The ABTS scavenging assay demonstrated that AAE had an antioxidant activity measuring 28.63 ± 1.02 TE µmol/mL. Assia Berrani et al. (2018) found that the ABTS = 16.01 ± 2.78 TE/g in a methanolic extract of the aerial part of *A. aretioïdes* [11]. Souad Senhaji et al. (2020) found values ABTS ranging between 0.69 ± 0.093 and 48.99 ± 1.31 µg TE/mg E as a result of the antiradical activities of aqueous and organic extracts of the aerial part of *A. aretioïdes* [1]. According to the total antioxidant capacity method, AAE had a phosphomolybdenum reduction of 117.29 ± 2.66 µg Asc. equivalent/mg extract.

Even though AAE has many antioxidant properties, we still need to compare extracts with different polarities, as well as with other antioxidant methods like iron chelation and ferric-reducing power.

A bivariate Pearson analysis (Table 3) was performed to investigate the correlation between antioxidants and their effectiveness in eliminating free radicals. The findings demonstrated a highly significant positive association between total phenolic content (TPC) and total flavonoid content (TFC), with a correlation coefficient of 0.998. In addition, average and negative relationships were found between the total condensed tannins (TCT) and TFC, with a correlation coefficient of −0.582, as well as between TCT and TFC, with a correlation coefficient of −0.535. The results show that the amount of AAE found in polyphenols from the aerial part of *A. aretioides* is consistent with flavonoids. In parallel with the increase in TPC and TFC, we found that TCT decreased.

The findings demonstrated a significant positive association between the TFC with β-carotene bleaching assay (β-CBA) and the total antioxidant capacity (TAC), with a correlation coefficient of 0.972 and 0.648, respectively, and a significant inverse relationship with ABTS IC 50 and DPPH IC_50_, with correlation coefficients of −1.000 and −0.995, respectively. Similarly, there was a significant positive correlation between the TPC with β-CBA and TAC, with correlation coefficients of 0.908 and 0.690, respectively, and a significant negative relationship with ABTS IC 50 and DPPH IC_50_, with correlation coefficients of −0.997 and −0.988, respectively. In contrast, there was a negative correlation between the TCT with β-CBA and TAC, with a weak correlation coefficient of −0.376 and a strong correlation coefficient of −0.990, respectively, and an average positive relationship with ABTS IC_50_ and DPPH IC_50_, with correlation coefficients of 0.602 and 0.657, respectively.

This result suggests that flavonoids and the polyphenolic compounds in the aerial part of *A. aretioides* are mainly responsible for antioxidant ability. In contrast, TCTs’ role in neutralizing free radicals in these extracts is diminished.

**Figure 2:**
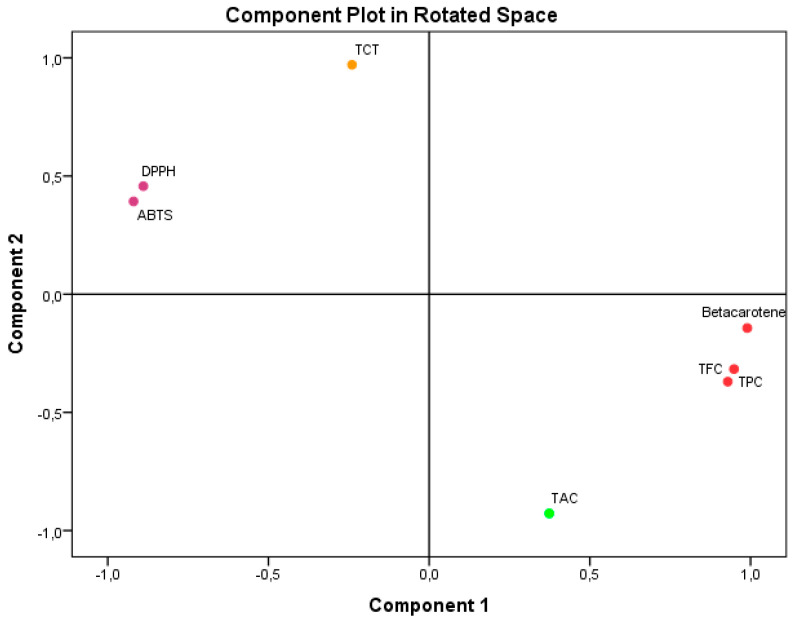
Principal Component Analysis (PCA) based on the different measurements of bioactive compounds and antioxidant activities in the *A. aretioides* hydroethanolic extract. DPPH, DPPH scavenging capacity IC50; β-carotene, β-carotene bleaching assay; ABTS, ABTS scavenging assay; TAC, total antioxidant capacity; TPC, total polyphenol content; TFC, total flavonoid content; TCT, total condensed tannins.

Principal Component Analysis (PCA), as seen in Figure 2, accounted for 100% of the overall variance. Specifically, component 1 explained 65.378% of the variance, while component 2 explained 34.622%. Component 1 exhibited a very high positive correlation with β-carotene (0.990), TPC (0.949), and TFC (0.929). It also showed a substantial negative correlation with ABTS IC_50_ (−0.981) and DPPH IC_50_. Component 2 exhibited a very significant positive correlation with TCT (0.971). Conversely, it showed a negative correlation with TAC (−0.928).

The results unequivocally illustrate the impact of the bioactive components found in AAE on the antioxidant activities that were examined. The study findings have demonstrated that flavonoids and polyphenols have an active role in the antioxidant activities, namely in terms of ABTS IC_50_, DPPH IC_50_, and β-CBA. These activities show a high negative association with ABTS IC_50_ and DPPH IC_50_ and a strong positive correlation with β-CBA. These results align with component 1 of PCA. The limited impact of TCT on these antioxidant activities is also apparent from its mild negative correlation with component 1 of PCA. The antioxidant activity of TAC was weakened by the interference of flavonoids and polyphenols, as evidenced by their low negative relationship with component 2 of PCA. Regarding TCT, its impact on TAC was minimal or nonexistent because of its positive correlation with component 2 of PCA. The reason for these results is that the TAC value is directly related to the presence of all the antioxidants in the extract.

### 3.3. HPLC-DAD Phytochemical Analysis of AAE

For the purpose of this investigation, the analysis was carried out in order to determine the different kinds of compounds that are found in AAE. According to Table 4, this particular extract was discovered to contain twelve distinct phytochemicals. Hydroxycinnamic acids derivatives included cinnamic, ferulic, p-coumaric, and sinapic acids. Flavonoids consisted of naringin, quercetin, 3-O-β-D-glucoside, rutin, and apigenin. In addition, hydroxybenzoic acid derivatives included syringic and vanillic acids. Within the phenolic aldehyde, only vanillin was representative. The last identified compound was dicarboxylic acid, which is known as succinic acid.

The predominant chemical compounds identified in the AAE were cinnamic acid, followed by sinapic acid and vanillin, which were considered the most prevalent chemicals.

Cinnamic acid, an organic compound with fragrant properties, is a prominent chemical constituent present in various plants, including *Cinnamomum cassia* (Chinese cinnamon) and *Panax ginseng*, as well as in fruits, whole grains, vegetables, and honey. Previous studies have indicated that cinnamic acid demonstrates antioxidant, antibacterial, anticancer, neuroprotective, anti-inflammatory, and antidiabetic characteristics. It also acts as a radical chain reaction terminator by contributing electrons that react with radicals, resulting in the formation of stable compounds. Additionally, it is employed as an aromatic component in toiletries, flavorings, cosmetics, and detergents. It can be synthesized through the enzymatic deamination of phenylalanine [63].

Sinapic acid is ubiquitous in the plant kingdom and is found in various fruits, vegetables, cereal grains, and oilseed crops, as well as in certain spices and medicinal plants. Consequently, it is a prevalent component of the human diet. Compounds derived from sinapic acid are distinctive in the Brassicaceae family. Sinapic acid exhibits antioxidant, antimicrobial, anti-inflammatory, anticancer, and anti-anxiety properties. 4-Vinylsyringol, derived from the decarboxylation of sinapic acid, is a powerful compound that exhibits strong antioxidant and antimutagenic properties. It effectively inhibits the development of cancer and the production of inflammatory cytokines. Sinapine, also known as sinapoyl choline, is classified as an acetylcholinesterase inhibitor and has potential for therapeutic use in the treatment of numerous diseases. Primarily owing to their antioxidative properties, these compounds have been proposed for potential application in food processing, cosmetics, and the pharmaceutical sector [64].

Vanillin, also known as 4-hydroxy-3-methoxybenzaldehyde, is the primary compound found in natural vanilla. It is a highly significant and extensively utilized flavoring substance on a global scale. Similar to other phenolic compounds with low molecular weight, vanillin has antioxidant and antimicrobial characteristics, making it a promising candidate for application as a food preservative. It exhibits activity against both Gram-positive and Gram-negative bacteria that cause food spoiling. Additionally, it has demonstrated efficacy against yeasts and molds in fruit purées and laboratory growth conditions. An issue arises from the potent taste of vanillin at the smallest inhibitory concentrations needed. However, this can be mitigated to some extent by utilizing it alongside other antimicrobials that work together synergistically. This approach reduces the required effective concentrations. Vanillin has been found to exhibit antimutagenic properties, as demonstrated by its ability to reduce chromosomal damage caused by methotrexate in the Chinese hamster V79 cell line [65].

### 3.4. Antibacterial Activity

Table 5 displays the outcomes of the antibacterial effectiveness of AAE using the disc diffusion method against four bacterial strains with the detection of MIC and MBC.

The results demonstrated that AAE has highly potent antibacterial activity, as substantiated by two items of evidence: The inhibition zone exceeded 14 mm by using reference drugs imipeneme (10 µg/disc) or amoxicillin (25 µg/disc) as the positive control. The antibacterial effects of AAE can be linked to the phenolic compounds included in this plant extract [66,67]. The antibacterial action of the phenolic compounds is attributed to the processes of polyphenols and flavonoids, which involve the sequestration of metal ions like iron and non-specific interactions such as the creation of hydrogen bonds with the proteins present in the cell walls and enzymes of bacteria [68].

The minimum inhibitory concentration (MIC) values show that AAE had the same values of 15 mg/mL against the two Gram-negative bacteria strains, *E. coli* and *P. aeruginosa*. Similarly, the AAE exhibited the same MIC of 7.5 mg/mL against the two Gram-positive bacterial strains, *S. aureus* and *E. faecalis*. According to this finding, Gram-positive bacteria are more susceptible to AAE than Gram-negative bacteria. This result is in line with other findings that have been reported in the literature by a number of studies [45]. Antibacterial substances are unable to pass through the intricate cell membrane of Gram-negative bacteria, preventing them from entering the bacterial cell. This restriction results from lipopolysaccharides’ strong hydrophilicity, which only permits tiny hydrophilic solutes to flow past the cell membrane’s porins [69].The minimum inhibitory concentration (MIC) is influenced by various factors, such as the botanical source and specific plant component used for extraction, the extraction process itself (including temperature and duration), the solvent employed, the quantity of active components extracted, and the specific microorganism targeted for inhibition by the extract [41].

The minimum bactericidal concentration (MBC) values of the AAE were equal to the MIC for Gram-negative *E. coli*, while the MBC value was twice the MIC value for Gram-positive *P. aeruginosa* and Gram-negative *S. aureus* and *E. faecalis*. This finding indicates the good bactericidal activity of AAE, specifically against *E. coli*. The MBC/MIC ratios were lower than 4 for all the bacterial strains targeted; therefore, the AAE was a strong bactericidal.

Antibiotic resistance arose soon after the discovery of the first antibiotic and has persisted as a significant public health concern ever since. Addressing antibiotic resistance in clinical environments remains difficult, especially with the emergence of superbugs, or bacteria that exhibit resistance to multiple medications, referred to as multidrug-resistant (MDR) bacteria. The swift emergence of resistance has necessitated ongoing efforts by researchers to discover new antimicrobial medicines to combat it, despite a diminishing pipeline of novel drugs. Recently, the emphasis of antimicrobial discovery has transitioned to plants, fungi, lichens, endophytes, and diverse marine sources, including seaweeds, corals, and other microorganisms, owing to their promising attributes. In recent years, interest in phytomedicines has increased, propelled by their beneficial attributes. Nonetheless, further investigation is crucial to comprehensively elucidate the mechanisms of action and confirm the safety of antimicrobial phytochemicals. The discussion will also encompass future potential for augmenting the application of plant secondary metabolites in the fight against antibiotic-resistant diseases [70].

### 3.5. Anticancer Activity Against Breast Cancer Cell Lines

Cancer is a multifaceted disease characterized by cells that are able to avoid death. Tumor cell proliferation and tissue infiltration are mainly caused by aberrant cellular proliferation and death [71]. Moreover, cancer arises as a result of alterations in genetic, epigenetic, and transcriptional elements [72,73]. In Africa, cancer represents around 10 to 20% of treated medical cases, and it is expected to roughly double by 2030 [74]. Currently, there are various types of cancer that are acknowledged, one of which is breast cancer (BC), which ranks as the third leading cause of mortality in women [31].

**Figure 3:**
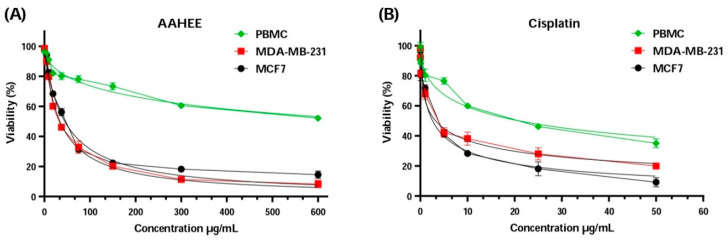
Cell viability of MCF-7, MDA-MB-231, and PBMC cells after 72 h of treatment with (**A**) AAE, *Anabasis aretioïdes* hydroethanol extract, and (**B**) cisplatin as a positive control using the MTT test.

Upon analyzing the results presented in Figure 3 and Table 6, the IC_50_ values for MDA-MB-231 and MCF-7 cell lines were close to the IC_50_ values of cisplatin, a standard chemotherapy drug. For MDA-MB-231, the concentration of AAE was 34.05 ± 1.03 µg/mL, which is higher than the 3.24 ± 0.37 µg/mL of cisplatin, and for MCF-7 it was 44.59 ± 3.33 µg/mL, higher than the 2.59 ± 1.30 µg/mL of cisplatin. However, the IC_50_ values of AAE and cisplatin were very different when it comes to the peripheral blood mononuclear cells PBMC, with 20.95 ± 4.19 µg/mL compared to 771.30 ± 12.30 µg/mL; this is what is considered selectively distinctive. The AAE selectivity index values that were found were much higher than those for cisplatin. This proves AAE’s distinction and effectiveness as a breast cancer treatment. The value for MDA-MB-231 was 22.65 ± 0.79, compared to 6.45 ± 1.48 for cispatlin. The results for MCF-7 showed a mean value of 17.30 ± 1.32, compared to 8.08 ± 4.41 for cispatlin. AAE’s efficacy and selectivity against the two types of breast cancer cell lines examined may be attributed to its polyphenol content. Polyphenols have the ability to interrupt or perhaps reverse cancer advancement by interacting with molecules in the intracellular signaling network that is responsible for cancer’s beginning and promotion. Polyphenols can trigger apoptosis in cancer cells by impacting a select few crucial elements of cellular transmission [75].

Consequently, there is a search for novel and safe therapies for cancer, some of which can be derived from natural sources [76]. The results suggest that AAE may have promising anti-cancer properties. This shows that more research is needed, such as mechanistic studies and in vivo trials, to confirm their effectiveness. Additional research into the possible use of these extracts in cancer treatment, coupled with a comprehensive understanding of their methods of operation, will greatly improve their practicality in a clinical setting. Moreover, the integration of plant extracts with other therapeutic modalities holds considerable promise in the collective effort to address a spectrum of microorganisms and critical ailments, including cancer. In this context, phytochemicals like flavonoids, polyphenols, alkaloids, saponins, terpenes, and other compounds found in plant extracts are known for their antioxidant, antimicrobial, and anti-cancer attributes [22,23,24].

### 3.6. In Silico Analysis of the Antioxidant, Antibacterial, and Anticancer Activity of the Components Found in AAE

NADPH oxidase (NOX) is a crucial enzyme involved in the generation of reactive oxygen species (ROS), and its function is closely regulated within cells. NADPH oxidase-generated reactive oxygen species (ROS) play a crucial role in protecting against pathogens and facilitating cellular communication. Many people think that oxidative stress, which is caused by NADPH oxidase producing too many reactive oxygen species (ROS), is the main reason why tissues become damaged in a number of respiratory inflammatory diseases and injuries, such as asthma, cystic fibrosis, acute respiratory distress syndrome (ARDS), and chronic obstructive pulmonary disease (COPD). Suppressing the activity of NADPH oxidase is a highly effective method to enhance the antioxidant activity and provide protection against diseases associated with oxidative stress [77]. NADPH oxidase inhibitors have the potential to preserve cellular well-being and restrain disease progression by reducing reactive oxygen species (ROS) generation, enhancing cellular antioxidant mechanisms, and mitigating chronic inflammation. Oxidative stress in COPD may be exacerbated by a decrease in endogenous antioxidants and inadequate consumption of dietary antioxidants. Oxidative stress is a major mechanism underlying COPD, contributing to chronic inflammation, cellular senescence, poor autophagy, diminished DNA repair, heightened autoimmunity, excessive mucus secretion, and compromised anti-inflammatory responses to corticosteroids. Oxidative stress consequently propels the pathogenesis of COPD and may accelerate disease progression, intensify exacerbations, and elevate comorbidities via systemic oxidative stress. This indicates that antioxidants may serve as effective disease-modifying therapies [78].

**Figure 4:**
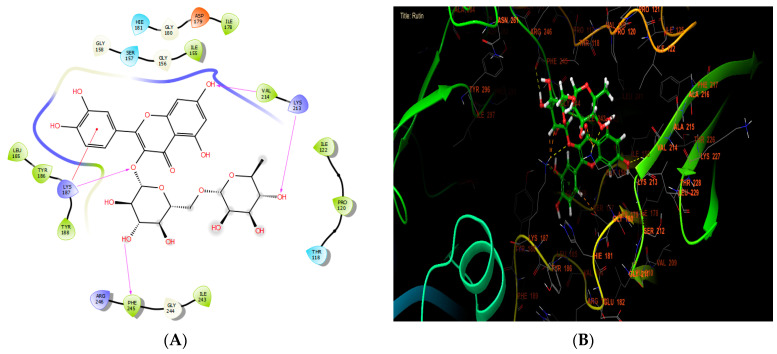
The ligand “rutin” interacts with the active site of NADPH oxidase through the 2D viewer (**A**) and the 3D viewer (**B**).

According to Table 7, our in silico research revealed that the three most active compounds against NADPH oxidase were rutin, quercetin3-O-β-D-glucoside, and succinic acid from leaf extract. These compounds had glide scores of −6.889, −6.788, and −6.692 Kcal/mol, respectively. In the active site of NADPH oxidase, rutin established four hydrogen bonds with residues VAL 214, LYS 213, PHE 245, and LYS 187, and a single Pi-cation bond with residue LYS 187 (Figure 4A,B).

Beta-ketoacyl-ACP synthase primarily functions to synthesize fatty acids of different lengths to be utilized by the organism. These applications encompass energy storage and the formation of cell membranes. Fatty acids have the ability to generate various compounds, such as prostaglandins, phospholipids, and vitamins, among other substances [79]. Beta-ketoacyl-ACP synthases are very important for creating lipoproteins, phospholipids, and lipopolysaccharides. Based on their potential benefits, they have become a focus for the development of antibacterial drugs. Bacteria modify the composition of their membranes by changing the phospholipids in order to adjust to their surroundings. Therefore, blocking this channel could serve as a strategic location for impeding the proliferation of bacteria [80].

**Figure 5:**
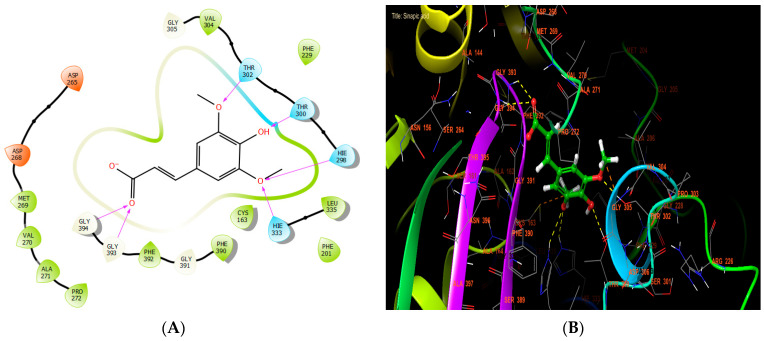
The ligand “ sinapic acid” interacts with the active site of beta-ketoacyl-[acyl carrier protein] synthase through the 2D viewer (**A**) and the 3D viewer (**B**).

Sinapic, syringic, and ferulic acids had the highest antibacterial efficacy against beta-ketoacyl-[acyl carrier protein] synthase from *E. coli*, with glide scores of −7.517, −6.636, and −6.558 Kcal/mol, respectively (Table 7). Sinapic acid established six hydrogen bonds with residues THR 302, THR 300, HIE 298, HIE 333, GLY 393, and GLY 394 in the protein active site (Figure 5A,B).

By transferring γ-phosphate from NTPs to NDPs and back, bacterial diphosphate kinase (Ndk) regulates the quantity of nucleoside triphosphate (NTP) in cells. Ndk is involved in protein histidine phosphorylation, DNA cleavage/repair, and gene control in addition to its main role in nucleotide metabolism. Ndk regulates the adaptive responses of bacteria as well. Ndks produced by intracellular bacteria block the function of numerous host defense mechanisms. These include inflammation, ROS production, phagocytosis, and cell apoptosis/necrosis. On the other hand, Ndks released by extracellular bacteria intensify the inflammatory response and cytotoxicity against host cells. Despite the differences in how Ndks from intra- and extracellular bacteria control host cellular activities, their primary purpose remains the same: They establish the host environment that facilitates colonization and spread [81]. As a result, inhibiting Ndk is an effective strategy for preventing the emergence of bacteria.

**Figure 6:**
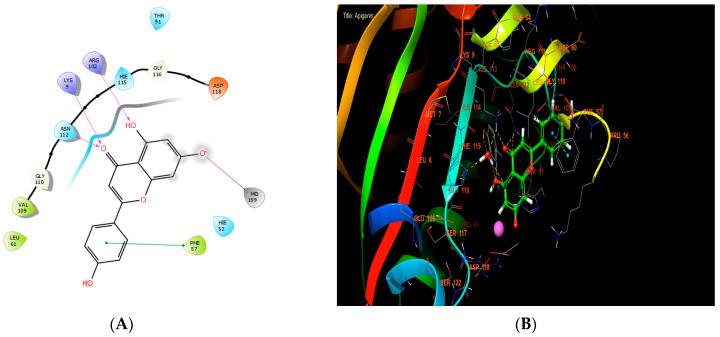
The ligand “apigenin” interacts with the active site of nucleoside diphosphate kinase through the 2D viewer (**A**) and the 3D viewer (**B**).

Apigenin, ferulic acid, and vanillin exhibited the highest levels of activity against *Staphylococcus aureus* nucleoside diphosphate kinase, with glide score values of −8.656, −7.933, and −8.174 kcal/mol, respectively (Table 7). In the active site of *S. aureus* nucleoside diphosphate kinase, apigenin established four hydrogen bonds with residues LYS 9 and ARG 102, and formed one salt bridge with MG 159 residue and one Pi-Pi stacking with residue PHE 57 (Figure 6A,B).

Caspase-3 is crucial in the process of fighting cancer by acting as a key player in the pathway of programmed cell death, known as apoptosis, which is necessary for the controlled removal of cancer cells. Caspase-3 is activated by initiator caspases in both the internal and extrinsic pathways of apoptosis. This activation results in the breakdown of essential cellular components and ultimately leads to cell death. Inducing caspase-3 activation can initiate apoptosis in cancer cells, hence counteracting their survival mechanisms and resistance to traditional treatment. In addition, some anticancer strategies focus on enhancing caspase-3 activity using drugs that either increase pro-apoptotic signals or inhibit anti-apoptotic proteins inside the tumor microenvironment. Therefore, utilizing the activation of caspase-3 is a feasible approach in cancer treatment, aiming to induce cell death specifically in cancerous cells while minimizing harm to healthy organs [82].

**Figure 7:**
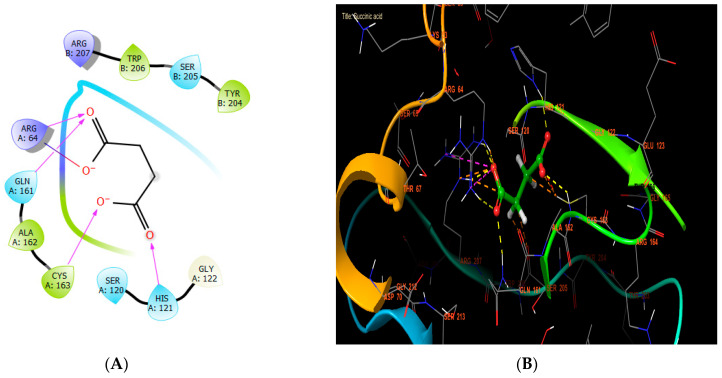
The ligand “succinic acid” interacts with the active site of caspase-3 through the 2D viewer (**A**) and the 3D viewer (**B**).

In our in silico study, succinic acid, rutin, and syringic acid were the most active molecules in the active site of caspase-3, with glide scores of −8.102, −7.003, and −6.722 kcal/mol (Table 7). Regarding that, succinic acid established four hydrogen bonds with the residues HIS A: 121, CYS A: 163, GLN A: 161, and ARG A: 64, and one salt bridge with residue ARG A: 64 (Figure 7A,B).

## 4. Conclusions

The findings have significant implications, particularly for the pharmaceutical and food industries. The varied chemical compositions and significant antioxidant and antibacterial properties included in AAE suggest its potential application as a natural supplement in nutritional and functional foods, and also in medical therapy. The hydroethanolic extract of the *Anabasis aretioides* aerial parts demonstrates a diverse range of chemical constituents and functional properties, such as antioxidant, antibacterial, and breast cancer-fighting capacities. These characteristics enhance the appeal of the extract for a multitude of applications. This experimental study presents novel data and facts that endorse the logical utilization of this indigenous medicinal plant found in the arid region of southeastern Morocco. This study should also strive for a thorough toxicological investigation, with the main goal being for the authors to discuss the results and how they can be interpreted from the perspective of previous studies and of the working hypotheses. The findings and their implications should be discussed in the broadest context possible. However, conducting an additional investigation is required to gather in vivo data and validate the findings of the activities. Future research directions may also be highlighted.

## Figures and Tables

**Table 1:** Total phenolic, flavonoids, and condensed tannins contents in AAE, from Figuig, Morocco.

Extract	Total Phenolic Content (mg GAE/100 g DW)	Flavonoid Content (mg RE/100 g DW)	Total Condensed Tannins (mg CE/100 DW)
AAE	159.32 ± 0.63	8.52 ± 0.32	8.73 ± 0.23

Data are presented as mean ± SD, and experiments were conducted in triplicate (n = 3). DW, dry weight; GAE, gallic acid equivalent; RE, rutin equivalent; CE, catechin equivalent.

**Table 2:** Free radical scavenging and antioxidant capacity of *A. aretioides* hydroethanolic extract (AAE).

Extract/Reference	DPPH Scavenging Capacity IC_50_ (µg/mL)	β-Carotene Bleaching Assay (mg/mL)	ABTS Scavenging (TE µmol/mL)	Total Antioxidant Capacity *
AAE	51.28 ± 0.91	4.44 ± 0.21	28.63 ± 1.02	117.29 ± 2.66
Ascorbic acid (AA)	8.10 ± 3.02	-	7.23 ± 1.37	-
Butylated hydroxytoluene (BHT)	-	0.132 ± 0.33	-	-

Values are expressed as mean ± SEM (n = 3), and experiments were conducted in triplicate (n = 3). * Total antioxidant capacity expressed as µg ascorbic acid equivalent/mg extract. TE: Trolox equivalent.

**Table 3:** Bivariate Pearson correlation analysis results regarding the relationship between antioxidants and their effectiveness in scavenging free radicals.

C	DPPH	β-Carotene	ABTS	TAC	TPC	TFC	TCT
DPPH	1.000						
β-carotene	−0.946	1.000					
ABTS	0.997	−0.966	1.000				
TAC	−0.756	0.503	−0.708	1.000			
TPC	−0.988	0.908	−0.997	0.648	1.000		
TFC	−0.995	0.972	−1.000	0.690	0.998	1.000	
TCT	0.657	−0.376	0.602	−0.990	−0.535	−0.582	1.000

Bivariate Pearson correlation analysis with a significance level of *p* < 0.01. DPPH, DPPH scavenging capacity IC_50_; β-carotene, β-carotene bleaching assay; ABTS, ABTS scavenging assay; TAC, total antioxidant capacity; TPC, total polyphenol content; TFC, total flavonoid content; TCT, total condensed tannins.

**Table 4:** HPLC-DAD bioactive compound profile of AAE.

Bioactive Compound		Chemical Formula	Classification	Retention Time	Area (%)
1	Syringic acid	C_9_H_10_O_5_	Hydroxybenzoic acid	4.831	2.62
2	Vanillic acid	C_8_H_8_O_4_	Hydroxybenzoic acid	5.901	0.8
3	Vanillin	C_8_H_8_O_3_	Phenolic aldehyde	6.176	13.3
4	Naringin	C_27_H_32_O_14_	Flavonoid	6.67	4.24
5	Cinnamic acid	C_9_H_8_O_2_	Hydroxycinnamic acid	7.213	29.39
6	Ferulic acid	C_10_H_10_O_4_	Hydroxycinnamic acid	8.608	6.84
7	p-coumaric acid	C_9_H_8_O_3_	Hydroxycinnamic acid	9.238	5.29
8	Sinapic acid	C_11_H_12_O_5_	Hydroxycinnamic acid	9.579	22.99
9	Succinic acid	C_4_H_6_O_4_	Dicarboxylic acid	10.732	0.43
10	Quercetin 3-O-β-D-glucoside	C_21_H_20_O_12_	Flavonoid	11.636	9.49
11	Rutin	C_27_H_30_O_16_	Flavonoid	12.364	3.6
12	Apigenin	C_15_H_10_O_5_	Flavonoid	14.856	1

**Table 5:** Results of the antibacterial activities of *A. aretioides* hydroethanol extract (AAE).

Bacterial Strains	Gram Type	AAE				Imipenem (10 µg/Disc)	Amoxicillin (25 µg/Disc)
		IZ * (mm)	MIC (mg/mL)	MBC (mg/mL)	MBC/MIC	IZ (mm)	IZ (mm)
*E. coli*	G-	25 ± 0.50	15	15	1	20 ± 0.50	15 ± 0.33
*P. aeruginosa*	G-	36 ± 2.66	15	30	2	25 ± 0.33	20 ± 0.20
*S. aureus*	G+	24 ± 1.50	7.5	15	2	18 ± 0.20	10 ± 1.30
*E. faecalis*	G+	27 ± 0.33	7.5	15	2	11 ± 0.66	9 ± 0.66

* IZ: inhibition zone; MIC: minimum inhibitory concentration; MBC: minimum bactericidal concentration; *S. aureus*, *Staphylococcus aureus* (ATCC 29213), *E. faecalis*, *Enterococcus faecalis* (ATCC 29212), *E. coli*, *Escherichia coli* (ATCC 25922), *P. aeruginosa*, *Pseudomonas aeruginosa* (ATCC 29212).

**Table 6:** IC_50_ values and selectivity indexes of *Anabasis aretioïdes* hydroethanolic extract (AAE) on cancer cell lines (MCF-7 and MDA-MB-231).

Treatments	IC_50_ Value ± SD (µg/mL) *			Selectivity Index **	
	MCF-7	MDA-MB-231	PBMC	MCF-7	MDA-MB-231
AAE	44.59 ± 3.33	34.05 ± 1.03	771.30 ± 12.30	17.30 ± 1.32	22.65 ± 0.79
Cisplatin	2.59 ± 1.30	3.24 ± 0.37	20.95 ± 4.19	8.08 ± 4.41	6.45 ± 1.48

* Values were obtained from three independent experiments and are expressed as means ± SD. ** Selectivity index = (IC_50_ of PBMC/IC_50_ of tumor cells).

**Table 7:** Glide score (Kcal/mol) based on the targeted oxidant or microbial protein and bioactive compounds of AAE.

Bioactive Compound of AAE	Glide Score (Kcal/mol)			
	NADPH Oxidase (PDB: 2CDU)	*E. coli* (PDB: 1FJ4)	*S. aureus* (PDB: 3Q8U)	Caspase-3 (PDB: 3GJQ)
Apigenin	−6.405	−6.488	−8.656	−5.832
Cinnamic acid	−4.637	−5.519	−7.053	−5.655
Ferulic acid	−5.401	−6.558	−7.933	−6.072
Naringin	−5.163		−4.674	−6.542
p-Coumaric acid	−5.017	−6.261	−7.678	−5.818
Quercetin3-O-β-D-glucoside	−6.788	−2.88	−6.226	−6.476
Rutin	−6.889	−4.837	−7.817	−7.003
Sinapic acid	−5.299	−7.517	−7.457	−5.901
Succinic acid	−6.692	−5.645	−8.251	−8.102
Syringic acid	−6.132	−6.636	−7.916	−6.722
Vanillic acid	−6.12	−6.217	−8.01	−6.516
Vanillin	−6.603	−6.471	−8.174	−5.392

## Data Availability

Data is contained within the article.
